# On the Morphological Characterization Procedures of Multilayer Hydrophobic Ceramic Membranes for Membrane Distillation Operations

**DOI:** 10.3390/membranes9100125

**Published:** 2019-09-23

**Authors:** Mohamed K. Fawzy, Felipe Varela-Corredor, Serena Bandini

**Affiliations:** Department of Civil, Chemical, Environmental and Materials Engineering (DICAM)-Alma Mater Studiorum, University of Bologna, I-40136 Bologna, Italy; mohamedkhaled.fawzy2@unibo.it (M.K.F.); felipe.varela2@unibo.it (F.V.-C.)

**Keywords:** hydrophobic ceramic membranes, multilayer membrane, morphological parameters, membrane characterization, gas permeance

## Abstract

The paper introduces some aspects of the characterization of hydrophobized multilayer ceramic membranes intended for use in membrane distillation (MD) operations. Four-layer hydrophobic carbon-based titania membranes, manufactured by the Fraunhofer Institute for Ceramic Technologies and Systems (IKTS, Hermsdorf, Germany), were tested according to the gas permeation technique. Gas permeance data were elaborated following the premises of the dusty gas model, to calculate the average pore size and the porosity-tortuosity ratio of each layer. Membrane testing was the opportunity to discuss which characterization method is more appropriate to obtain the membrane parameters necessary for the simulation of membranes in MD processes. In the case of multilayer membranes, the calculation of the morphological parameters should be performed for each layer. The “layer-by-layer gas permeation” method, previously introduced by other authors and completed in this work, is more appropriate for obtaining representative parameters of the membrane. Conversely, the calculation of morphological parameters, averaged over the entire membrane, might lead to heavy underestimations of the total membrane resistance and then to a heavy error on the transmembrane flux simulation.

## 1. Introduction

Since the beginning of the 1980s it has been well known that membrane distillation (MD) for aqueous solutions can operate with membrane contactors (MC) containing hydrophobic macroporous membranes [1,2]. Membranes are required with an average pore diameter in the range from 20 to 200 nm, manufactured with hydrophobic materials able to guarantee minimum liquid entry pressure (*LEP_min_*) values at room temperature not lower than nearly 1 bar, to have a safety margin for industrial applications at higher temperatures. Membranes with the smallest thickness as possible are recommended in order to increase the diffusion rate across the membrane. 

Recently, there has been a growing interest among the scientific community in the application of ceramic membranes for MD, owing to their high thermal and chemical stability; such materials might give greater morphological stability than polymeric membranes over time [3].

Ceramic membranes are typically asymmetrical, formed by the deposition of different layers made of γ-alumina and/or titania and/or zirconia and/or silica or a combination of them. The main drawback for their application in MD is their hydrophilicity; it can be circumvented by the modification of the top layer by polymer grafting (generally fluoroalkylsilanes) and/or carbonization techniques. Patents [4,5] and many papers document innovations and advances in the hydrophobization of ceramic membranes, both in the shape of flat samples and of hollow fibers; the most representative documentation of the state-of-the-art is given by [6,7,8,9,10,11,12,13,14,15,16,17,18]. 

Typically, in each paper, authors introduce their own manufacturing procedure, both of the basic ceramic membrane and of the grafting procedure, and present results on the characterization of the membrane material (scanning electron microscope (SEM) pictures, contact angle, mercury porosimetry, thermogravimetric analysis (TGA), gas permeation tests, liquid entry pressure (LEP) with water at room temperature, and/or gas-liquid displacement tests). Sometimes the characterization is extended to the measurement of water flux across the membrane performed in one of the MD operation modes; however, of the case study, no process simulation nor the corresponding mathematical modeling nor the final comparison with the experimental results are reported.

Since the possibility of manufacturing hydrophobic ceramic membranes is well-established, nowadays it is important to understand which characterization techniques are fundamental to assess firstly the process applicability of a membrane and, secondly, to define the separation efficiency of a module.

The process applicability of a membrane can be tested by the *LEP_min_* measurement performed in a wide range of operating temperatures, or by the minimum liquid entry temperature measurement performed in a wide range of differential pressures across the membrane. The normalized-flooding-curve method, recently presented and discussed by Varela-Corredor and Bandini in [19] and properly developed for ceramic membranes, can be followed.

The separation efficiency of a module can be predicted by good process simulation.

Obviously, the process principle in the case of ceramic membranes is exactly the same as in the case of polymeric ones. As far as the pressure difference across the membrane does not overcome the breakthrough pressure (*LEP_min_*), a liquid-vapor interface is immobilized at the pore mouth. The liquid vaporizes and vapors diffuse across the membrane under a driving force of partial pressure difference, which is strictly related to the gradient of the main operation variable (an activity gradient and/or a pressure gradient, as an example [1,2]). When asymmetric multilayer ceramic membranes are used, the liquid–vapor interface should be located on the top layer of the membrane (the lower pore size layer), and diffusion occurs across all the different layers of the membrane. 

Therefore, we can expect that the equations describing the mass transfer across the ceramic membrane are the same as in symmetric polymeric membranes, with the complication that they should be discretized for each layer and properly combined to calculate the overall mass transfer coefficient of the membrane.

The main features of the mass transfer equations have been known since the end of the last century [20,21,22,23,24,25,26]; a review of MD modeling in polymeric membranes has recently been reported in [2]. In order to calculate the mass transfer coefficient of the membrane, all the equations require knowledge of a few morphological parameters, such as the thickness and the porosity–tortuosity ratio. Those parameters are not adjustable parameters, since they can be determined independently of flux measurements in MD operations. Typically, in symmetrical polymeric membranes, they are calculated by elaborating gas permeation data according to the dusty gas model (DGM) equations, whereas for polymeric asymmetrical membranes the same method can be used to obtain information on the skin morphology (details in chapter 8 of [2]). In [27] an improved method was developed for asymmetrical polyvinylidene fluoride (PVDF) membranes, later applied by the same authors to the characterization of ceramic membranes also [10,11], to obtain averaged parameters over the entire membrane. Only Weyd et al. [28] performed the characterization of multilayer membranes according to a “layer-by-layer” technique, which allowed morphological parameters of each single layer to be obtained by the elaboration of gas permeation data according to the DGM.

The purpose of this work is to present a critical discussion on the results obtained from the morphological characterization of multilayer hydrophobized ceramic membranes, following both the conventional protocol developed for polymeric membranes and the protocol derived by the application of the layer-by-layer technique. The study is developed by using hydrophobic carbon-based ceramic membranes manufactured by the Fraunhofer Institute for Ceramic Technologies and Systems (IKTS, Hermsdorf, Germany) as cylindrical membranes, in the shape of tubular single-channels and capillary bundles. Gas permeation tests are performed, followed by *LEP_min_* measurements carried out according to the normalized-flooding-curve method [19]. For the purposes of this work, the *LEP_min_* measurements had as the only objective to test if the membranes had the requirements for process applicability for MD.

After a preliminary introduction of the basic equations describing the mass transfer in MD across a multilayer cylindrical membrane, the procedure for calculating the morphological parameters of multilayer cylindrical membrane is described, according to the principles of the DGM. The work is completed with a critical discussion of the results.

## 2. Materials and Methods 

### 2.1. Membranes and Modules 

**Figure 1:**
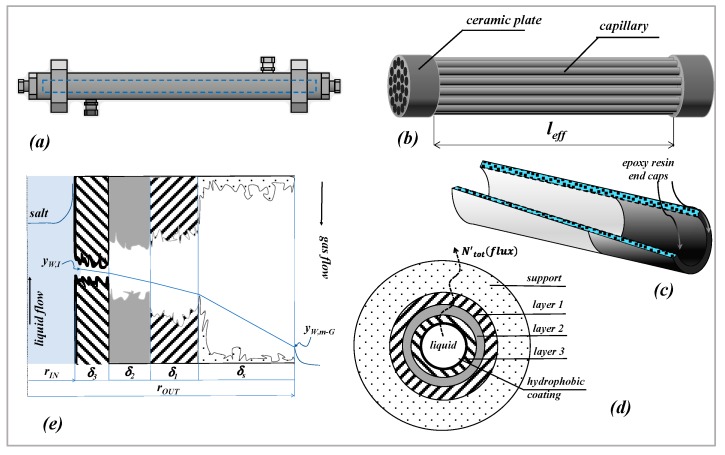
Scheme of membrane and module. (**a**) housing; (**b**) capillary bundle; (**c**) section of a cylindrical membrane; (**d**) multilayer membrane cross section; (**e**) detail of the radial section of the coated membrane showing the water composition profiles, with reference to SGMD of a salt-water solution.

Hydrophobic carbon-based titania membranes were used. Membranes were supplied by the Fraunhofer Institute for Ceramic Technologies and Systems (IKTS, Hermsdorf-Berlin, Germany) in the shape of single channels and of capillaries; capillaries were arranged in unbaffled bundles and used in a housing according to a shell and tube configuration. The schemes of the housing, of a bundle and of a single membrane (single channel or capillary) are reported in Figure 1. 

According to the information given by the manufacturer, the basic ceramic membrane (indicated in the following as “uncoated” samples) is the same both in the case of single channels and of capillaries; the only difference is the “support” thickness. The uncoated membrane is a 4-layer membrane of different pore sizes and thicknesses. The “support” is the outer layer (4500 nm pore size), followed by intermediate layers tagged as “layer 1” and “layer 2” (30 μm thickness each, 800 and 250 nm pore size, respectively); the top layer is the “layer 3” (10 μm thickness and 100 nm nominal pore size) located at the lumen-side.

The manufacturing technique of the basic membrane was reported in [29] and most of the morphological characterization of each layer was documented in detail in [28], where SEM pictures are shown also; manufacturing of capillaries and bundles was described in [30]. The hydrophobization procedure was described in detail by the manufacturer also; it consists of two subsequent techniques: the “uncoated” 4-layer membrane is firstly carbon-coated by the deposition and pyrolysis of a polymeric precursor (as patented in [4] and also documented in [31,32]) and, secondly, is surface-grafted with fluoroalchylsilane (FAS, tridecafluoro-1,1,2,2-tetra-hydro-octyl-trichloro-oxysilane) as patented in [5].

Each cylindrical membrane (both “uncoated” and “coated”) was refined by epoxy resin endcaps (Figure 1c) to prevent leakages of liquids or gases across the annulus at the inlet section, from the lumen side towards the outer side. The same epoxy resin was used by the manufacturer to seal each capillary on the ceramic plates of the bundle and to make the ceramic plates impermeable

In the case of a perfect hydrophobic coating, when the membrane is used in MD operations, the liquid–vapor interface is immobilized at the pore entrance of the “layer 3” and the membrane can operate in no-wetting conditions. Only the coating located on the lower porosity layer, the “layer 3”, is the actual barrier of the membrane. The scheme of the generic membrane section is reported in Figure 1d).

#### 2.1.1. Single Channels

The geometrical characteristics of single channels were the following: 7 mm inner diameter, 10 mm outer diameter; 250 mm total length, with an effective length of 224 mm, excluding the end caps length; effective inner area A_IN_ = 49.2 cm^2^.

Different kinds of samples were tested:

“support A/B” = uncoated membranes containing only the layer “support”;

“S-DA”, “S-DB” = uncoated 4-layer membranes;

“S2515, S2516” = coated 4-layer membranes.

The complete information of the morphological parameters of each layer of the uncoated single channels was given by the manufacturer, except for “layer 3”; they are reported in Table 1 as nominal values. Those nominal values derived from a dedicated measurement procedure explained by the manufacturer and reported in [28]. Each layer was characterized according to SEM observations (to estimate the thickness), mercury porosimeter (to estimate the volume porosity) and gas permeation tests (to calculate the tortuosity factor). Firstly, measurements were performed starting by a membrane containing only the “support”; secondly, “layer 1” was deposited and the same kind of measurements were performed; the procedure was repeated also for the “layer 2”, sequentially. After each measurement step, the authors calculated the unknown tortuosity factor of the specific layer by the elaboration of gas permeation data according to the DGM equations, using the thickness, the pore diameter and the porosity values of all the other layers determined at the previous steps.

It is necessary to observe that the above sequence of measurements is important. The “layer-by-layer” method allowed to get the characterization of all the open pores, involved in the gas transfer across the multilayer membrane. However, the results should be interpreted and used in the correct way: the values of the tortuosity factors are not absolute; rather, they are strictly dependent on the volume porosity and on the pore diameter values inserted in the equations of the DGM used for their calculation. As a consequence, that elaboration technique gives the overall evaluation of the porosity-tortuosity ratio (ε/τ) parameter, rather than the evaluation of the single value of each term. For that reason, only the (ε/τ) parameters are reported in Table 1.

#### 2.1.2. Capillary Bundles

The geometrical characteristics of the coated bundles are reported in Table 2, in which the dimensions of the corresponding capillaries are indicated also. It is worth noting that the manufacturer informed that all the morphological parameters of the “uncoated” bundles were corresponding to those of the single-channel case (see Table 1—case a), except for the support thickness. Also, for the bundle cases, neither the pore diameter nor the porosity-tortuosity ratio of “layer 3” was available.

### 2.2. Characterization Procedures 

For any virgin sample, gas permeation measurements were performed at room temperature and, sequentially, the samples underwent *LEP_min_* measurements according to the normalized flooding curve method [19]. For the purposes of this work, the results of *LEP_min_* measurements are aimed only at testing if the membranes have the requirements for the process applicability for MD.

Gas permeation was carried out with dried air at 20 or 24 °C, whereas the *LEP_min_* was measured with demineralized water (3–14 μS/cm at 25 °C) at 25 or 60 °C. 

For gas permeation tests, a quite traditional equipment was used [2,27]. Volumetric gas flow rates were measured across the membrane and/or the bundle in dead-end mode, by keeping a pressure difference across the membrane. All samples were tested in “straight mode”, driving the flux from the lumen side to the outer side; some of them were also tested in “reverse mode”. The pressure difference across the membrane was varied in the range from Δ*P_tot_* = 0.1 to 1.2 bar, whereas the corresponding arithmetic mean of the pressure values (*P_av_*) were varied in the range from 1 to 7 bar. The volume flow rates were measured by flowmeters upstream of the membrane, in the case of flow rates higher than 1200 STP m^3^/h, whereas a flowmeter was used downstream of the membrane in the case of flow rates from 400 to 1200 STP m^3^/h.

For each pressure value, a minimum of 10 min stabilization time was required after the regulation of the setpoint; at least three subsequent measurements were performed, the arithmetic mean is reported as the final value. The protocol of measurement required a preliminary drying of the virgin sample in an oven at 60 °C (4 h long) followed by a stabilization step in which the sample was kept, for a minimum of 1 hour, at a pressure difference of 1 bar, with the atmospheric pressure downstream of the membrane.

On presenting the results, in order to compare different samples with different interfacial areas, the overall membrane permeance is reported with reference to the inner area of the sample, as indicated in Equation (1) (*N_air_* is the molar flow rate). Data are plotted along the average pressure values (*P_av_*).

(1) $$\mathrm{permeance}=\frac{N_{\mathrm{air}}}{A_{\mathrm{IN}}\Delta P_{\mathrm{tot}}}$$

## 3. Theoretical Premises for the Results Discussion

In order to understand the “results and discussion” section, some theoretical premises are necessary.

### 3.1. Diffusive Mass Transport in Membrane Distillation (MD) 

Mass transport in MD across a multilayer cylindrical membrane is here considered, in which the driving force is represented by a composition gradient across the membrane. The scheme and notation of Figure 1e are used for the development of the following equations; in that case the composition gradient is kept by a sweeping gas downstream of the membrane [1,2]. Reference is made to the simple case of aqueous solutions containing only one volatile compound (as, for example, in the case of sodium chloride-water solutions with air as a gas stream). However, the same kind of composition profile across the membrane can be expected in other MD operations, such as direct contact MD and/or air gap MD and/or osmotic distillation, for example [1,2]. It is worth-noting that in this problem conceptualization we are interested in describing mass transfer across the membrane, assuming that the composition value at the feed/membrane interface (y_WI_) is a known value. However, the complete modelling should certainly account for the mass and heat transfer in the external phases which determine the value of y_wI_

The membrane is cylindrical and composed of four layers (indicated as *j*); each layer is characterized by its corresponding pore diameter (*d_pj_*), thickness (*δ_j_*) and porosity-tortuosity ratio ${\left(\varepsilon /\tau \right)}_{j}$. Although the hydrophobic coating is in principle distributed along the surface of all the pores, only the coating located at the inner surface of the membrane (the “layer 3”) is effective to give the hydrophobicity necessary to immobilize a liquid–vapor interface at the feed-membrane side. The concept is explained in Figure 1e) in which all the pores are depicted as unflooded by the liquid feed. As a consequence, in the case of a perfect coating, mass transfer across the membrane is represented by the transfer of water vapor across four cylindrical layers containing a stagnant gas phase.

The case of molecular diffusion across a stagnant layer under steady-state conditions is well known in literature [33]. In the case of macroporous membranes, those equations require some modifications in order to account for possible different contributions to the diffusive mechanism, as well as of the number of pores existing in the cylindrical wall of the membrane. 

Although molecular diffusion is the main mechanism, owing to the rather high pore sizes, the contribution of Knudsen diffusion can be accounted for also, and included in an equivalent diffusivity ($D_{Weq}$), according to the Bosanquet equation [34], as represented by Equation (2). The Knudsen diffusivity in Equation (2) depends on the average pore diameter and on the average temperature existing in the *j*-layer; *R_g_* and *M_W_* represent the universal gas constant and the molecular weight, respectively. $D_{WG}$ is the molecular diffusion of water in the gas phase, which should be calculated at the temperature and pressure existing in the *j*-layer.

(2) $$\frac{1}{D_{Weq,j}}=\frac{1}{D_{WG}}+\frac{1}{D_{W,Kn,j}}\;;\;D_{W,Kn,j}=\frac{d_{pj}}{3}\sqrt{\frac{8R_{g}T_{j}}{\pi M_{W}}}$$

The number of pores per unit length (${N^{\prime }}_{p}$) is included in the volume porosity (*ε_j_*) which generally depends on the radial coordinate. It can be considered as an average value, according to the relationships (3), which are written referring to the case of cylindrical pores of equal diameter, assuming the logarithmic mean surface as average interfacial area:

(3) $$\varepsilon_{j}(r)≃\varepsilon_{av}=\frac{V_{void}}{V_{tot}}=\frac{N_{p}\pi d_{pj}^{2}\times \delta_{j}}{4A_{LM,j}\times \delta_{j}}=\frac{N_{p}d_{pj}^{2}}{8r_{LM,j}L}=\frac{{N^{\prime }}_{p}d_{pj}^{2}}{8r_{LM,j}}$$

The general equation describing the total molar flow per unit length across a single membrane layer (${N^{\prime }}_{tot}$) is then represented by Equation (4), in which the mass transfer coefficient of water in the *j*-layer of the membrane ($k_{Wj}$) is defined straightforwardly, accounting for the volume porosity and for the pore tortuosity:

(4) $${N^{\prime }}_{tot}=\frac{2\pi \varepsilon_{j}c_{j}D_{Weq,j}}{\tau_{j}\times ln\frac{r_{OUT,j}}{r_{IN,j}}}ln\left(\frac{1-y_{W,r_{OUT,j}}}{1-y_{W,r_{IN,j}}}\right)=k_{Wj}c_{j}2\pi r_{LM,j}\times ln\left(\frac{1-y_{W,r_{OUT,j}}}{1-y_{W,r_{IN,j}}}\right)\;;\;k_{Wj}={\left(\frac{\varepsilon }{\tau }\right)}_{j}\frac{D_{Weq,j}}{\delta_{j}}$$

$r_{IN,j}$ and $r_{OUT,j}$ represent the inner and outer radius of the layer (*j)*, *c_j_* is the molar concentration of the gas phase inside the layer and *y* is the mole fraction.

In the case of a multilayer membrane, under steady state conditions, by using Equation (4) it is easy to combine the mass transfer coefficients of each layer to obtain the mass transfer coefficient of the membrane ($k_{Wm}$); with reference to the scheme and notation of Figure 1e), Equation (5) can finally be derived:

(5) $$\frac{{N^{\prime }}_{tot}}{k_{Wm}c_{m}2\pi r_{LM,m}}=ln\left(\frac{1-y_{W,m-G}}{1-y_{W,I}}\right)\;;\;\frac{1}{k_{Wm}c_{m}2\pi r_{LM,m}}=\sum_{j=1}^{3,s}\frac{1}{k_{Wj}c_{j}2\pi r_{LM,j}}$$

Equation (5) does not contain adjustable parameters depending on the process type nor on the process fluids; the morphological parameters of each layer can be determined by independent measurements, as made in the “layer-by-layer method” by Weyd et al. [28], for instance. When the parameters are known, Equation (5) can be used to predict total flux across the membrane, in order to simulate module and process performances at any operative conditions of temperature, pressure and composition, once the mole fraction of water is related to the corresponding bulk conditions.

### 3.2. Pressure-Driven Gas Transport in Macro-Porous Membranes 

Gas permeation across porous solids is typically due to the contribution of three main mechanisms [35]: a viscous motion according to a Poiseuille flow, the Knudsen diffusion and the so-called slip flow regime. For macro-porous membranes such as those of this work, the simpler dusty gas model [36] can be used with a good approximation accounting only of the viscous and Knudsen contributions expressed with the same equations. In addition, since this work continues and completes the morphological characterization started in [28], it is important to apply the same equations used in that paper (the DGM), since all the morphological parameters (reported in Table 1 as nominal ones) derive from that: they are related to each other and, therefore, they should be used consistently.

A summary of the equations used in this work is reported in the following (schemes and notation of Figure 1 are used).

At a generic axial section of a layer *j* of a cylindrical membrane, the steady-state gas flow rate of *i*-compound across the layer per unit length (${N^{\prime }}_{i}$), under a constant pressure difference ($\Delta P_{j}$), can be expressed as reported in Equation (6a).

(6a) $$\begin{array}{l} {N^{\prime }}_{i}=\alpha_{ij}\;2\pi r_{LM,j}\;\Delta P_{j}\\ \alpha_{ij}={\left(\frac{\varepsilon }{\tau \delta }\right)}_{j}\left[a_{ij}P_{av,j}+c_{ij}\right]={\left(\frac{\varepsilon }{\tau \delta }\right)}_{j}\left[\frac{d_{pj}^{2}}{32\eta_{G}R_{g}T_{j}}P_{av,j}+\frac{4}{3}\frac{d_{pj}}{\sqrt{2\pi R_{g}T_{j}M_{i}}}\right]\\ P_{av,j}=\frac{P_{IN,j}+P_{OUT,j}}{2};\Delta P_{j}=P_{IN,j}-P_{OUT,j} \end{array}$$

where $\alpha_{ij}$ is the permeance of *i*-compound across the *j*-layer; $P_{IN,j}$ and $P_{OUT,j}$ are the inlet and outlet pressures, respectively, in the *j*-layer; $a_{ij}$ and $c_{ij}$ are coefficients representing the viscous and Knudsen diffusion contributions, respectively; $\eta_{G}$ is the dynamic viscosity of the gas phase. 

In the case in which the parameters can be assumed as constant values along the membrane length *L* (or as average values along *L*), the total gas flow rate across the layer can be written as:

(6b) $$N_{i}=\alpha_{ij}\;2\pi r_{LM,j}\;L\;\Delta P_{j}=\alpha_{ij}\;A_{LM,j}\;\Delta P_{j}$$

Apparently, the permeance of each layer depends on the morphological parameters of the layer, on the gas properties and on the operative conditions. 

Combining the resistances in all the layers, the overall membrane permeance of the *i*-compound ($\alpha_{im}$) can be expressed by Equation (7):

(7) $$\begin{array}{l} N_{i}=\alpha_{im}\;A_{LM,m}\;\Delta P_{tot}\Leftrightarrow N_{i}\left[\sum_{j=1}^{3,s}\frac{1}{\alpha_{ij}A_{LM,j}}\right]=\Delta P_{tot}\\ \frac{1}{\alpha_{im}A_{LM,m}}=\sum_{j=1}^{3,s}\frac{1}{\alpha_{ij}A_{LM,j}};\Delta P_{tot}=\sum_{j=1}^{3,s}\Delta P_{j}=P_{IN,3}-P_{OUT,s} \end{array}$$

It is important to observe that the porosity parameter of each layer, appearing in Equations (6), corresponds to the same volume porosity defined in Equation (3), owing to the same geometry of the problem.

In the case of a 4-layer membrane, as is the case in this work, Equations (6) represent a set of equations which allow us to calculate the gas molar flow and the pressures along each layer, if the operative conditions of temperature and of *P_IN,3_* and *P_OUT,s_* (that is of $\Delta P_{tot}$) are given and the values of the morphological parameters of each layer are known. The permeance of each single layer, as well as the total membrane permeance, can be finally estimated straightforwardly.

On the other hand, Equations (6) to (7) can be used also to calculate the unknown morphological parameters of a layer, by fitting the experimental results of total permeance, assuming that the morphological parameters of all the other layers are known. 

That procedure is exactly the one used by Weyd et al. in [28] to calculate the tortuosity factors of the “support” and of the “layers 1 and 2” of the “uncoated” single channels, and it will be used also in this work to estimate the morphological properties of “layer 3”, both for uncoated and for coated samples.

The results can then be compared to those obtained following the usual protocols used for polymeric membranes [2,10,27], according to which average values of the porosity–tortuosity ratio *(ε/τ)_m_* and of the average pore diameter ($d_{pm}$) are calculated by fitting the experimental results of permeance over the entire membrane. In that case, referring to the notation of this work, Equation (8) represents the overall membrane permeance as a function of the average pressure across the membrane, according to the DGM premises.

(8) $$\begin{array}{ll} \alpha_{im}=\frac{N_{i}}{A_{LM,m}\;\Delta P_{tot}} & ={\left(\frac{\varepsilon }{\tau \delta_{tot}}\right)}_{m}\left[a_{im}P_{av}+c_{im}\right]\\ & ={\left(\frac{\varepsilon }{\tau \delta_{tot}}\right)}_{m}\left[\frac{d_{pm}^{2}}{32\eta_{G}R_{g}T_{av,m}}P_{av}+\frac{4}{3}\frac{d_{pm}}{\sqrt{2\pi R_{g}T_{av,m}M_{i}}}\right]\\ \Delta P_{tot}=P_{IN}-P_{OUT}\;;\; & P_{av}=\frac{P_{IN}+P_{OUT}}{2}\;;\;\delta_{tot}=\sum_{j=1}^{3,s}\delta_{j} \end{array}$$

It is self-evident that Equation (8) predicts a linear behavior of the total membrane permeance with the average pressure existing across the overall membrane; the parameters can be estimated according to a very simple fitting procedure which accounts for the slope and the intercept of the interpolating straight line.

## 4. Results and Discussion

### 4.1. Single Channels

**Figure 2:**
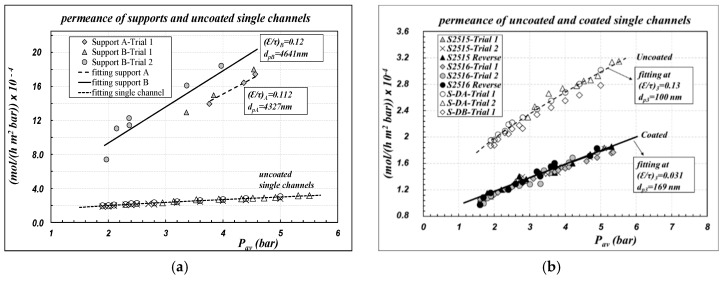
Air permeance of single channels at 20 °C, along the average pressure across the membrane. (ΔP_tot_ = 0.4–1.2 bar). Symbols = experimental data; lines = fitting by Equations (6) and (7). Parameters are collected in Table 1.

Air permeance data, referring to the inner area of the sample as defined in Equation (1), are reported in Figure 2 for different kinds of single channels, as a function of the average pressure across the membrane. Figure 2a compares the permeance of uncoated “support” with the overall permeance of the “uncoated” 4-layer membrane. The permeance of the “support” is nearly five to 10 times greater than the corresponding values for the uncoated 4-layer membranes, owing to its very large pore size. Figure 2b compares the permeance of the “uncoated” samples with the corresponding values obtained for “coated” samples. No differences are observed between the results in the “straight mode” and in the “reverse mode”; this result is an intrinsic confirmation that the measurement procedure is good to characterize the “open pores”. Conversely, there is a remarkable effect of the coating, resulting in a 50% decrease in the permeance of the “coated” samples with respect to the “uncoated” ones. In Figure 2a,b, experimental data are also compared with the results of the fitting procedure (lines), performed according to Equations (6) and (7). All the parameters obtained are collected in Table 1 and compared with the corresponding nominal values given by the manufacturer; the quality of the fitting is documented as percentage relative error (%RE) between calculated and experimental data.

The main results are the following. 

Apparently, the typical linear behavior of the DGM is obtained, both for uncoated and for coated samples, thus indicating that the model premises are fulfilled. There was a substantial reconfirmation of the nominal values of the “support”. As regards “layer 3” of uncoated samples, a reconfirmation of the pore diameter value declared by the manufacturer was obtained, whereas the (ε/τ) value was obtained lower than the corresponding values of “layers 2 and 1”, but aligned with them.

The procedure was repeated for “layer 3” of “coated” samples, assuming that the morphological parameters of the supporting layers (“layer 2”, “layer 1” and “support”) were not affected by the coating procedure, owing to their higher pore size. The results are rather interesting: the (ε/τ) value was obtained rather lower than the “uncoated” case, whereas an increasing value of the average pore diameter was calculated. Those results are not in contradiction with each other, rather, they can be explained by accounting for the fact that the first step of the coating procedure, the carbonization, can cause pore-blocking of the thinner pores. Of course, also the grafting procedure with fluoroalkylsilanes can lead to an additional decrease of the pore size. Owing to both of the motivations, the pore size distribution can change and shift the average size towards greater values. Those results are mostly aligned also with what was observed by other authors with different membranes grafted by fluoroalckylsilanes [10,12,37].

**Figure 3:**
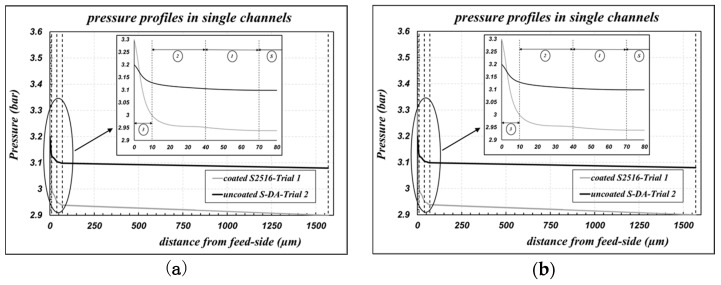
Pressure profiles along the radial coordinate of the membrane; elaborations of permeance data according to Equations (6) and (7). (**a**) Data from Figure 2b at *P_av_* = 3.1 bar (parameters in Table 1—case b); (**b**) data from Figure 4 at *P_av_* = 3.3 bar (parameters in Table 3—case a).

While performing data fitting, the pressure profiles along the radial coordinate of the membrane can be obtained also. The cases of the “uncoated S-DA” and of the “coated S2516” samples are reported in Figure 3a. We can observe that very steep pressure drops are obtained across the “layer 3”, which is the less gas permeable layer: the membrane permeance is mainly controlled by the properties of “layer 3”.

As a final calculation, all the experimental data reported in Figure 2b were elaborated to estimate the average membrane parameters, according to the usual protocols for polymeric membranes, as reported in Equation (8). Results are again collected in Table 1 as “average membrane values”, both for the cases of “uncoated” and “coated” samples.

Also, by those calculations, the coating effect in the pore reduction or in the pore-blocking is self-evident. However, the values of porosity-tortuosity ratios seem to be rather inconsistent with their physical meaning, since they are obtained as numbers greater than unity. Those values, however, are aligned also with those obtained by other authors who performed elaborations of the same kind. As an example, basing on the results documented by Koonaphapdeelert and Li [10], it is possible to calculate a value of ε/τ = 1.88 for ungrafted 300 μm thickness alumina fibers and ε/τ = 2.24 after their grafting.

We suspect that the membrane parameters averaged over the entire membrane are not representative of the membrane properties. A more detailed and critical discussion about the meaning of these results is reported in Section 4.3.

Finally, the *LEP_min_* measurements gave the threshold values of 2.7 and 2.3 bar at 60 °C for the samples S2515 and S2516, respectively; those values certainly represent a positive test of the process applicability of the membranes, which can be proposed as good elements for membrane contactor devices in the future.

### 4.2. Capillary Bundles

**Figure 4:**
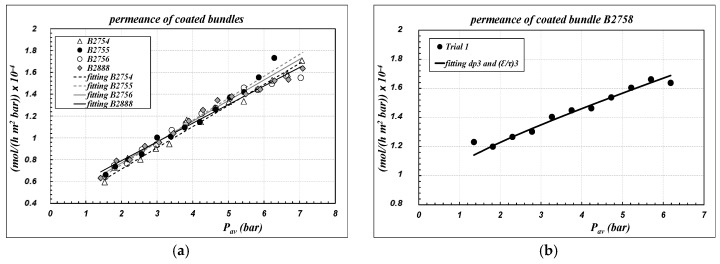
Air permeance of bundles at 24 °C, along the average pressure across the membrane. (ΔP_tot_ = 0.09–0.54 bar). symbols = experimental data; lines = fitting by Equations (6) and (7). Parameters are collected in Table 3.

Air permeance data of “coated” bundles, referring to the inner area of the sample as defined in Equation (1), are reported as symbols in Figure 4, as a function of the average pressure across the capillaries. Experimental data are compared with the results of the fitting procedure, performed according to Equations (6) and (7) to calculate the parameters of “layer 3”, assuming that the morphological parameters of the other supporting layers were not affected by the coating procedure. The results are collected in Table 3. The corresponding pressure profiles across the membrane for the bundle B2758 are reported in Figure 3b also, as an example of the general trend. 

There is a substantial reconfirmation of the results already obtained for single-channel membranes, with regards to the linear trend of data as well as to the range of the permeance values. Indeed, since “layer 3” is the controlling layer on the gas permeance, the overall air permeance is not affected in a sensible manner by the different thicknesses of the “support” layer of the single-channel and of the capillary, as could have been expected. The reproducibility of the results is very good for all the bundles; the sample B2758, however, seems to be much more aligned with single channels.

For the bundles B2754, B2755, B2756 and B2888, it can be observed that after coating there is a remarkable increase of the pore diameter of “layer 3”, in association with a remarkable decrease of the porosity–tortuosity ratio. That result is again consistent with the hypothesis of pore-blocking of the thinner pores, but it should alert about the process applicability of the membranes, since we might expect low values of the breakthrough pressure. Also, for the bundles, experimental data reported in Figure 4 were elaborated to calculate the average membrane parameters according to the definitions reported in Equation (8). Results are reported in Table 3 as “average membrane parameters”.

Finally, the *LEP_min_* measurements gave the threshold values of 6.9 and 5.8 bar at 25 °C for the samples B2758 and B2756, respectively; those values are notably higher than those typically measured for polymeric membranes of similar pore size [2,19]. The process applicability test of those membranes has been considered as positive.

### 4.3. Discussion and Conclusions

The morphological characterization developed in the previous sections allowed us to obtain the parameters (*d_pj_*) and (*ε/τ*)*_j_* necessary for the determination of the overall mass transfer coefficient of the membrane, as described by Equations (4) and (5), to be used to simulate/predict the transmembrane flux in MD operations. 

Based on those results, it was then possible to estimate the mass transfer resistances of each layer and the overall resistance across the multilayer membrane. To that purpose, the relationships reported in Equation (5) are re-elaborated according to Equation (9) in order to obtain an expression of the mass transfer resistance of water across the total membrane (${\bar{R}}_{Wm}$) and across each layer *j* (${\bar{R}}_{Wj}$).

(9) $$\;{\bar{R}}_{Wm}=\frac{1}{k_{Wm}}=\sum_{j=1}^{3,s}{\bar{R}}_{Wj}=\sum_{j=1}^{3,s}\frac{r_{LM,m}}{k_{Wj}\;r_{LM,j}}$$

**Figure 5:**
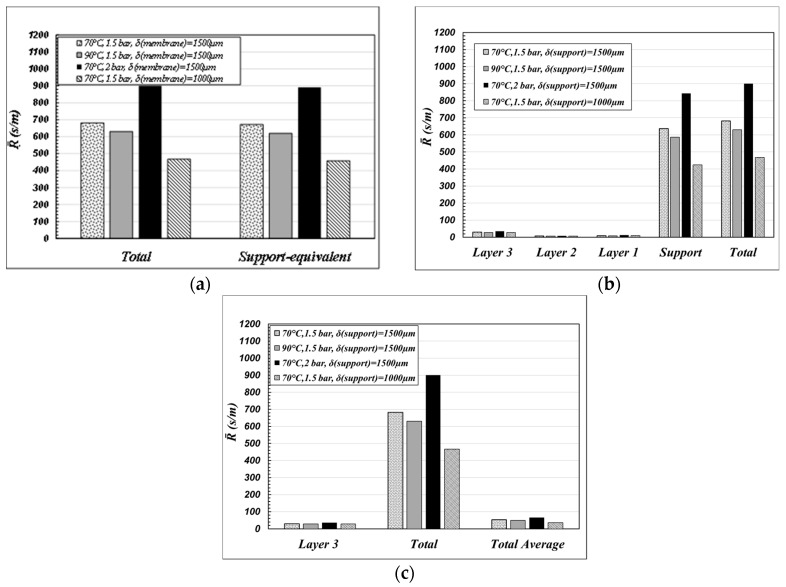
Mass transfer resistance of water vapor in air across each layer of a coated single channel (Equation (9)). Comparison among different calculation methods at various operative conditions and different support thicknesses (parameters from Table 1). (**a**) Resistance of each single layer and their sum resulting in total membrane resistance indicated by *“Total*”. (**b**) Membrane resistance on considering the contributions of the four layers indicated by *“Total”* and the resistance on regarding the membrane composed of a single layer having the morphological properties of the support layer indicated by *“Support-equivalent”.* (**c**) Resistance of layer 3, resistance of the membrane on considering the contributions of the four layers indicated by “Total” and the resistance of the membrane on considering average morphological values of the whole membrane (Table 1—case (c)) indicated by “*Total Average*”

The case of water-air is considered. The water mass transfer resistances were calculated at 70 °C and 90 °C, at 1.5 and 2 bar in the gas phase, for “support” thicknesses of 1500 and 1000 μm, referring to the case of “coated” single channels. The results are collected in Figure 5.

The results plotted in Figure 5a put clearly in evidence that, when the membrane is used in MD operations, the “support” is the controlling step of the mass transfer resistance across the membrane. Since the mass transfer is mainly due to a diffusive mechanism (Equation (4)), the thinner is the “support” thickness the greater is the total flux across the membrane. By contrast, “layer 3” is not remarkable in determining the mass transfer. It actually works only as a mere support for the hydrophobic coating, which is to be deposited/grafted on thin pores in order to prevent pore-wetting (that is to give the highest values of *LEP_min_* as possible). In order to increase the total transmembrane flux in MD operations, it is necessary to decrease the “support” thickness as much as possible, by keeping the pore size of “layer 3” as it is, in order to be sure to immobilize the liquid–vapor interface on it.

The role of the “support” is put in evidence in Figure 5b also, in which the total membrane resistance of Figure 5a is compared with the resistance calculated according to Equation (9) as “support-equivalent”, that is as the membrane parameters were coincident with those corresponding to the “support” at the total thickness of the membrane ($\delta_{tot}$).

The results reported in Figure 5c are interesting and remarkable. That figure compares the total water resistance calculated by using the parameters of each layer (“Total” is as in Figure 5a) with the overall water resistance calculated by using the “average membrane values” (“Total Average”), as defined in Equation (8) and reported in Table 1—case c. Apparently, the use of the morphological parameters averaged over the entire membrane gives a heavy underestimation of the overall membrane resistance with respect to the calculation performed by using the morphological parameters of each single layer.

Since in all the MD operations the mass transfer across the membrane is mainly a diffusive transfer, it is important to have a very good estimation of the porosity-tortuosity ratio, in order to be able to simulate the transmembrane flux. For the membranes here considered, the calculation of *(ε/τ)_m_* as an average value, basing on gas permeation data, is not practicable, since it is greatly affected by the determining role of “layer 3” on the air permeance, which, by contrast, is not so important when the membrane operates in MD. Absurdly, an error in the evaluation of the parameters of “layer 3” could not be so remarkable in determining the overall mass transfer of the membrane. Also, errors in the estimation of the pore diameter of “layer 3” are not so relevant, since the pore diameter is only necessary to calculate the Knudsen contribution to the equivalent diffusivity, which typically has a minor role (with the exception of vacuum membrane distillation). Conversely, a precise evaluation of the parameters of the “support” is fundamental, since it is the controlling layer on the mass transfer resistance of the membrane.

As a consequence, the method of fitting permeance data with average morphological parameters over the entire membrane, as was generally suggested in the case of polymeric symmetric membranes [2] and as was generally performed also in the case of ceramic membranes [3,10], should be used carefully, since it might lead to heavy errors on the simulation of membrane flux in most of the MD operations.

The calculation of the morphological parameters of each layer, according to the “layer-by-layer” gas permeance method, as introduced in [28] and completed in this work, is more appropriate.

The final verification of these conclusions will be reported in the next paper in which experimental data of fluxes across the membrane will be compared with the predicted values at the same operative conditions calculated by using the morphological parameters obtained in this work. 

Finally, it is interesting to observe that many other kinds of hydrophobic membranes have been proposed in literature such as composite membranes [38] and/or mixed matrix membranes used for pervaporation [39,40]. In those cases, however, the “layer-by-layer method” of combining the resistances according to the DGM should be modified significantly, since the gas permeance is regulated also by different transport mechanisms, and more specific detailed studies should be performed.

**Notation**

$$\%RE=\frac{1}{N_{data}}\sum_{q=1}^{N_{data}}\left|\frac{\alpha_{\exp,q}-\alpha_{cal,q}}{\alpha_{\exp,q}}\right|\%\;=\;\mathrm{percent}\;\mathrm{relative}\;\mathrm{error}$$

## Figures and Tables

**Table 1:** Morphological parameters of single channels. Nominal values are compared with those obtained by gas permeation tests elaborated according to different procedures (the number of trials is reported in brackets).

Layer	*δ_j_* (μm)	*dp_j_* (nm)	*ε_j_*	*(ε/τ)_j_*	%RE
**Uncoated Single Channels**					
support	1500 ^a^	4500 ^a^	0.33 ^a^	0.11 ^a^	-
	-	4484 (3) ^b^	-	0.11 (3) ^b^	12.16
layer 1	30 ^a^	800 ^a^	0.34 ^a^	0.20 ^a^	-
layer 2	30 ^a^	250 ^a^	0.39 ^a^	0.34 ^a^	-
layer 3	10 ^a^	100 ^a^	-	-	-
		100 (3) ^b^	-	0.13 (3) ^b^	3.52
average membrane values	1570 ^a^	232 (3) ^c^	-	3.90 (3) ^c^	2.39
**Coated Single Channels**					
layer 3	10 ^a^	169 (6) ^b^	-	0.031 (6) ^b^	3.54
average membrane values	1570 ^a^	259 (6) ^c^	-	1.94 (6) ^c^	2.98

a: nominal values by manufacturer; b: calculated by Equations (6) and (7); c: calculated by Equation (8).

**Table 2:** Characteristics of “coated” capillary bundles.

Single Capillary			Bundle and Vessel			
Code	*δ*_Support_ (μm)	ID/OD (mm)	N_f_	L_eff_ (cm)	A_IN_ (cm^2^)	Shell OD (cm)
B2754	750	1.56/3.20	37	20	363	3.60
B2755						
B2756						
B2758	580	1.90/3.20	22	20	263	2.50
B2888	750	1.90/3.54	37	20	442	3.60

N_f_ = number of fibers; ID = inner diameter; OD = outer diameter.

**Table 3:** Capillary bundles: elaborations of gas permeance data.

Bundle	Parameters of Layer 3 ^a^			Average Membrane Parameters ^b^		
Code	d_p3_ (nm)	*(ε/τ)_3_*	RE%	dp_m_ (nm)	*(ε/τ) _m_*	RE%
B2754	548	0.0029	3.14	468	0.27	3.36
B2755	534	0.0032	4.69	1232	0.053	7.56
B2756	435	0.0044	3.41	354	0.44	3.40
B2888	328	0.0069	3.13	337	0.38	3.25
B2758	68	0.084	3.03	87	3.414	2.89

a calculated by Equations (6) and (7); b calculated by Equation (8).
